# Gene alternation of cerebrospinal fluid in patients with leptomeningeal metastases of lung adenocarcinoma using next-generation sequencing

**DOI:** 10.1186/s12885-022-09597-y

**Published:** 2022-05-25

**Authors:** Hainan Yang, Lei Wen, Yingying Pan, Changguo Shan, Weiping Hong, Hui Wang, Cheng Zhou, Linbo Cai, Caicun Zhou

**Affiliations:** 1 Department of Oncology, Shanghai Pulmonary Hospital &Thoracic Cancer Institute, School of Medicine, Tongji University, Shanghai, 200092 China; 2 Department of Oncology, Guangdong Sanjiu Brain Hospital, Guangzhou, 510510 China; 3 Department of Medical Oncology, Shanghai Pulmonary Hospital & Thoracic Cancer Institute, School of Medicine, Tongji University, No. 507, Zheng Min Road, Shanghai, 200433 China

**Keywords:** Leptomeningeal metastases, Cerebrospinal fluid, Genotyping, Lung adenocarcinoma, Epidermal growth factor receptor (EGFR) tyrosine kinase inhibitor

## Abstract

**Background:**

Epidermal growth factor receptor tyrosine kinase inhibitors (EGFR-TKIs) provide a better prognosis in *EGFR*-mutant non-small cell lung cancer (NSCLC). Nevertheless, the outcome of leptomeningeal metastasis (LM) remains poor. In addition, due to limited access to intracranial tumour tissue, gene alterations associated with leptomeningeal metastasis from lung adenocarcinoma (LM-LUAD) are unclear.

**Methods:**

Forty-five patients with LM-LUAD from May 2019 to June 2021 in Guangdong Sanjiu Brain Hospital were enrolled in this study. Seventy-five percent (34/45) of patients with LM harbored *EGFR* mutations, and patients with progressive disease (PD) of LM had 3rd-generation EGFR-TKI therapy and were defined as Cohort 1; those without 3rd-generation EGFR-TKI therapy were defined as Cohort 2. Next-generation targeted panel sequencing (NGS) was performed in each cerebrospinal fluid (CSF) sample of the two cohorts, and 9/45 LM-LUAD patients had matched plasma (PLA).

**Results:**

The common gene alterations discovered in the CSF of LM-LUAD were *EGFR* mutation (34/45, 75%), *TP53* (25/45, 56%), *CDKN2A* (9/45, 20%), *ALK* (7/45, 16%), *CTNNB1* (6/45, 13%), *MET* (5/45, 11%), *APC* (4/45, 9%), *FGF4* (4/45, 9%), *FGF3* (4/45, 9%), *ERBB2* (4/45, 9%), and *PIK3CG* (4/45, 9%). Cooccurring mutations of *TP53* and *EGFR* were found in 49% (22/45) of patients and correlated with poor prognosis. *CDKN2A* was identified in 20% (9/45) of patients and presented slightly shorter overall survival (OS) than those without (7.1 versus 8.8 months, *p* = 0.2). Cohort 1 had more genes associated with poor prognosis, consisting of *CDK4*, *CDKN2A*, *PIK3CG*, or *PIK3CA*, and *YES1* and *MET* were more likely to be detected in cohort 2. The alteration of *EGFR* was comparable between CSF and matched PLA. Incidences of gene alterations such as *CDK4*, *CDKN2A*, *MET*, *SOX2*, *JAK2*, *BRAF*, and *PIK3CG* were more likely to be identified in CSF. All mutant allele frequencies (MAF) were much higher in CSF than in matched PLA.

**Conclusions:**

CSF could be a potential candidate for the genetic profiling of LM-LUAD, demonstrating the genetic characteristics of LM in *EGFR*-mutated lung adenocarcinoma on diverse EGFR-TKI therapies.

## Introduction

Lung cancer is one of the leading causes of cancer-related death [1]. The discovery of oncogenic genes, such as epidermal growth factor receptor (*EGFR*), anaplastic lymphoma kinase (*ALK*), and *ROS1,* has changed the therapeutic approach for selected non-small cell lung cancer (NSCLC). Epidermal growth factor receptor tyrosine kinase inhibitors (EGFR TKIs) are effective in treating patients whose tumours harbor sensitive *EGFR* mutations. However, the response rate varies among those people. Leptomeningeal metastasis (LM) is still a significant risk factor, and approximately 10% of advanced NSCLC patients with *EGFR* mutations are diagnosed with LM [2]. LM is a detrimental complication of NSCLC and is related to poor prognosis [3]. Although somatic genetic modification has been firmly established, the potential genetic alteration that accounts for the development of intracranial metastasis is still unclear. Additional efforts have revealed that cerebrospinal fluid (CSF) is close to intracranial metastasis and is more representative of brain lesions than plasma (PLA) for detecting relevant mutations [4], which makes CSF an alternative option to trace the evolution of the tumour genome. Next-generation targeted panel sequencing (NGS) panels could detect variants in multiple relevant genes and reveal the changes in percentage mutant allele frequency (MAF) with sufficient CSF samples, which could shed light on the unknown driver gene.

## Methods

### Patient characteristics

We retrospectively screened 200 consecutive patients diagnosed with lung adenocarcinoma at Guangdong Sanjiu Brain Hospital from May 2019 to June 2021. Among these patients, forty-five patients diagnosed with LM who underwent NGS based on CSF were ultimately included in the present study. LM was diagnosed based on typical central nervous system symptoms, i.e., headache, dizziness, etc., combined with either tumour cells identified in CSF samples or positive results in brain magnetic resonance imaging (MRI). All CSF samples were obtained through lumbar puncture with approximately 10 mL CSF collected for NGS testing. Among these 45 patients, nine also had paired PLA, and 8 ml plasma was collected for NGS simultaneously.

All patients signed informed consent, and the research protocol was approved by the Research Ethics Committee of Guangdong Three Nine Brain Hospital.

### Library material

Cell-free DNA (cfDNA) from CSF and whole blood was extracted using the QIAamp DNA FFPE Tissue Kit (Qiagen) according to the manufacturer’s protocol. Next-generation target sequencing of CSF was mainly performed using the 168-gene panel. The preparation of cfDNA in CSF and the NGS sequencing library were fully described in previous research [5]. NGS samples were prepared according to a standard protocol, and sequencing data were mapped to the human genome.

### Statistical analyses

Patient demographic information was reported using descriptive statistics, and Fisher’s exact test was used to analyse categorical variables. Overall survival (OS) curves were calculated using the Kaplan–Meier method from the diagnosis of leptomeningeal metastases to death or the last follow-up date. Hazard ratios were calculated with the use of a Cox proportional hazard model. *P* < 0.05 was deemed statistically significant. Statistical analyses and curves were generated with SPSS 21 and GraphPad Prism 6 software.

## Results

### Clinical characteristics of patients with LM-LUAD

The median age was 53 (range from 30 to 73 years), and 55.6% (25/45) of the patients were female in this study. In addition, 2% (1/45) of the patients had wild-type, 7% (3/45) patients had *ROS1* fusions, and 16% (7/45) of patients had *ALK* fusions. The majority of patients in this study harbored *EGFR* mutations, accounting for 75% (34/45) (Table 1).

**Table 1:** Patients and tumour characteristics of the study cohort

Characteristics	No. (%)
Median Age, years (range)	53 (30–73)
Sex	
Female	25 (55.6)
Male	20 (44.4)
Pathology	
Adenocarcinoma	45 (100)
Gene mutation status	
EGFR	34 (75)
EGFR-L858R	22 (49)
EGFR-19Del	9 (20)
GFR-L861Q	2 (4)
EGFR-20ins	1 (2)
ALK	7 (16)
ROS1	3 (7)
wild-type	1 (2)
Diagnosis of LM	
Positive cytology in CSF	25 (56)
Typical imaging in brain MRI	20 (44)
Matched plasma specimen	9 (20)
Survival	
Yes	5 (11)
No	40 (89)

*LM* Leptomeningeal Metastasis. *EGFR* Epidermal Growth Factor Receptor. *ALK* Anaplastic Lymphoma Kinase. *CSF* Cerebrospinal Fluid. *MRI* Magnetic Resonance Imaging

### Gene landscape of 45 CSFs from LM-LUAD

**Fig. 1:**
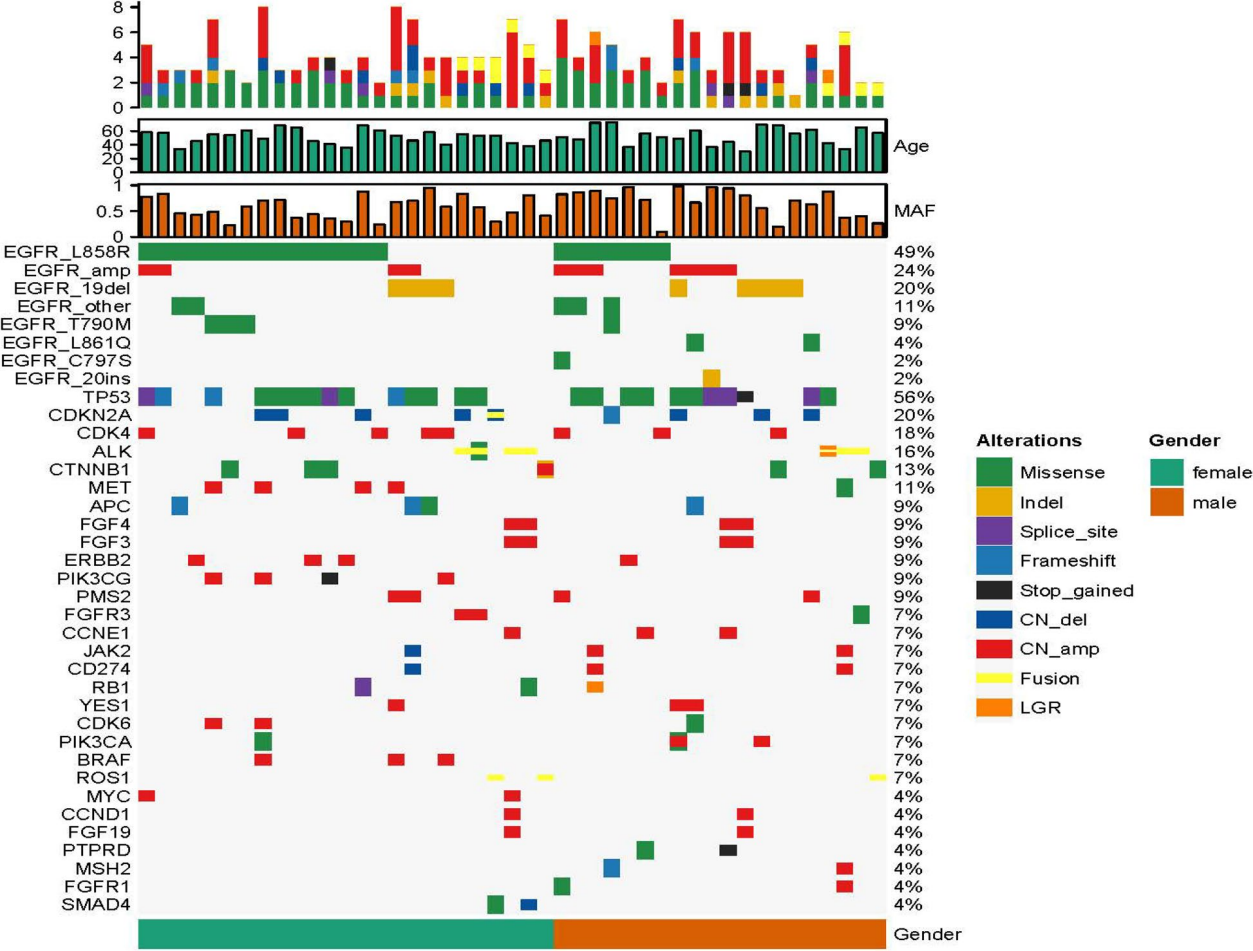
Gene landscape of 45 CSFs from LM-LUAD. Oncoprint of the multiple genetic alterations from the 45 cases is shown. The three top bars indicate the number of mutations, age, and MAF in each patient. The left side-bar presents names of the detected genes in CSF, and the right side-bar demonstrates the number of specific mutations in patients. Colour keys represent the variant type of the relative gene. MAF mutant allele frequency. CSF: cerebrospinal fluid

Here, we performed NGS on 45 CSF specimens from LM-LUAD to characterize the genomic mutation during the development of LM, and a handful of genetic profiles of CSF cfDNA were present (Fig. 1).

*EGFR* and *TP53* are the two most frequently mutated genes in CSF samples. The most frequent mutation of *EGFR* demonstrated that *EGFR*-L858R, *EGFR*-19Del, *EGFR*-20ins, and EGFR-L861Q were 49, 20, 2, and 4%, respectively. *TP53* was detected in 25 (25/45, 56%) patients, and coexisting *TP53* and *EGFR* were found in 22 (22/45, 49%). *CDKN2A* was the most frequent copy number variation (CNV); copy number deletion was detected in 7 patients, copy number deletion together with gene fusion in 1 patient, and frameshift variant identified in 1 patient. *CDK4* copy number gain was recognized in 8 patients (8/45, 18%), and *MET* copy number variation was captured in 5 patients (5/45, 11%). In addition, *ERBB2*, *PIK3CG*, and *PMS2* copy number variations were identified in 4 patients, and *JAK2* copy number variations were detected in three patients. Other potential metastasis genes were also captured in CSF cfDNA, including *YES1, CDK6*, *PIK3CA*, *BRAF*, and *ROS1*, which were seen in 3 patients, and *PTPRD*, *MSH2*, *FGFR1*, and *SMAD4*, which were captured in 2 patients.

### OS associated with the presence or absence of related genes

**Fig. 2:**
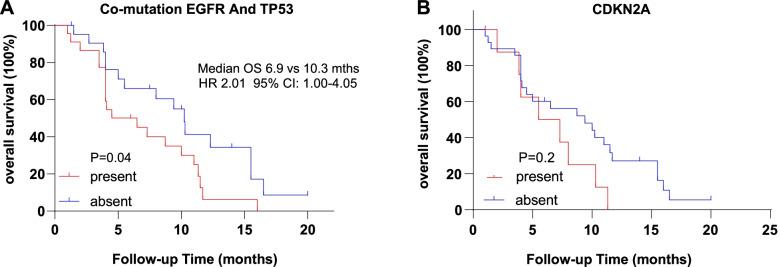
OS associated with the presence or absence of related genes. Kaplan–Meier curves of patients with or without relative genes. **A**, with or without co-mutation of *EGFR* and *TP53*. **B**, presence or absence of *CDKN2A*. OS was defined as the time from diagnosis of leptomeningeal metastasis to death or the last follow-up time. *p* values were calculated using a two-sided log-rank test. Hazard ratios were calculated with the use of a Cox proportional hazard model. *EGFR* denotes epidermal growth factor receptor. *TP53* denotes the tumour protein P53. OS overall survival. CI confidence interval. HR hazard ratio

Coexisting *TP53/EGFR* showed shorter OS than the opposing group (6.9 versus 10.3 months, *p* = 0.04) (Fig. 2A). In our findings, the second most common coexisting gene mutation identified was *ALK* fusions together with *TP53*, which was discovered in 2/45 (4.4%) patients who had an OS and progression-free survival (PFS) of 2 and 7.5 months on crizotinib, respectively. Taking the limited number into consideration, there was no comparison of OS benefit results in our study. *CDKN2A* was identified in 9/45 (20%) patients in our research. Accumulate evidence demonstrated that *CDKN2A* is common in cerebrospinal fluid (CSF) samples of lung adenocarcinoma patients with central nervous system (CNS) metastases, and concurrent *CDKN2A* with *EGFR*-sensitive mutations indicated inferior median intracranial PFS (iPFS). However, in our research, this known genetic alteration was not correlated with a worse prognosis compared to those without such an alteration (Fig. 2B).

### Specific genetic signatures of the two subgroups

**Fig. 3:**
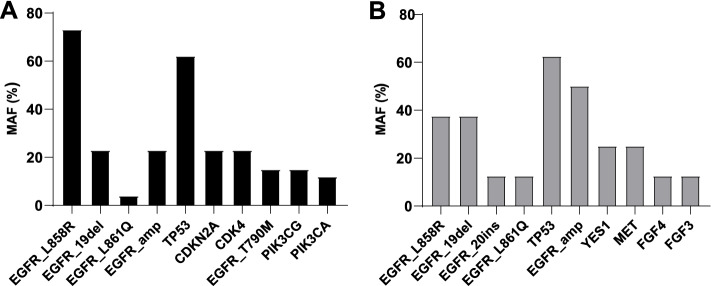
Bar charts show the distribution of the top 10 genes detected in each CSF sample in the 2 cohorts. **A** Cohort 1, PD of LM with 3rd generation EGFR-TKI therapy. **B** Cohort 2, PD to LM without 3rd generation EGFR-TKI therapy

The top 10 genes varied among the different cohorts (Fig. 3, Table 2).

**Table 2:** The top 10 genes detected in each cohort

Cohort 1 No. (%)		Cohort 2 No. (%)	
No. of patients *N* = 26		No. of patients *N* = 8	
EGFR-L858R	19 (73)	EGFR-L858R	3 (37.5)
EGFR-19del	6 (23)	EGFR-19del	3 (37.5)
EGFR-L861Q	1 (4)	EGFR-20ins	1 (12.5)
EGFR-amp	6 (23)	EGFR-L861Q	1 (12.5)
TP53	16 (62)	TP53	5 (62.5)
CDKN2A	6 (23)	EGFR-amp	4 (50)
CDK4	6 (23)	YES1	2 (25)
EGFR-T790M	4 (15)	MET	2 (25)
PIK3CG	4 (15)	FGF4	1 (12.5)
PIK3CA	3 (12)	FGF3	1 (12.5)

*TP53* was the most frequent alteration among the two subgroups, with detection rates of 62% (16/26) and 62.5% (5/8) in cohorts 1 and 2, respectively. *EGFR* amplification was the common gene in those two cohorts, with detection rates of 23% (6/26) in cohort 1 and 50% (4/8) in cohort 2. Unlike the discovered genes in cohort 1, *MET*, *YES1*, and *FGF3/FGF4* copy number amplification were more likely present in cohort 2, which indicated the different pathways involved in the progressive disease of LM on various EGFR-TKI treatments. *EGFR* mutation (EGFRm) T790M was identified in 15% (4/26) of patients in cohort 1 and was not detected in cohort 2.

### Concordance of EGFR-activating mutations in CSF samples with matched PLA

**Fig. 4:**
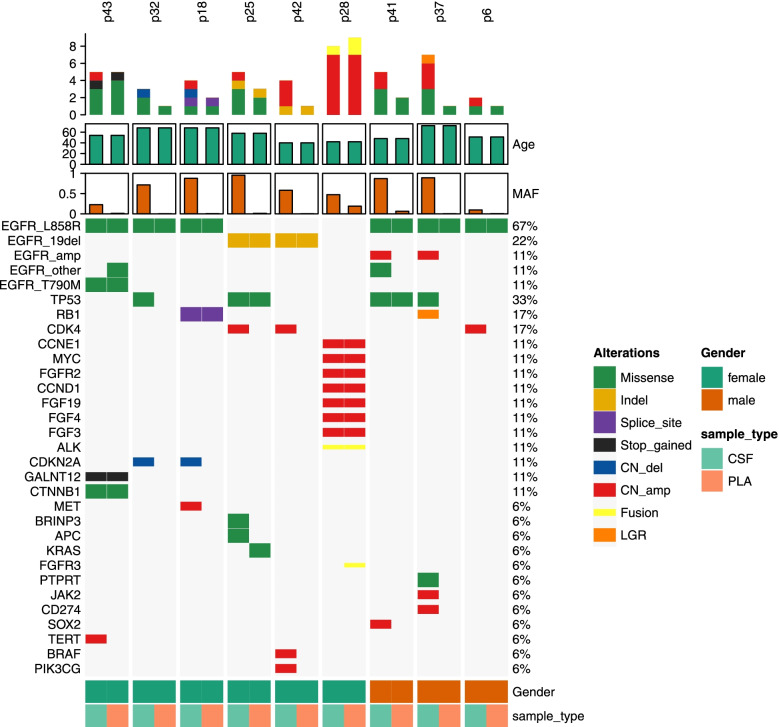
Concordance of EGFR-activating mutations in CSF samples with matched PLA. Oncoprint of the multiple genetic alterations from the 9 matched CSF-PLA cases are shown. The three top bars indicate the number of mutations, age, and MAF in matched samples. The left side-bar presents names of the detected genes in matched CSF-PLA pairs, and the right side-bar demonstrates the number of specific mutations in patients. Colour keys represent the variant type of the relative gene. MAF: mutant allele frequency. CSF: cerebrospinal fluid. PLA plasma

Our study demonstrated that sensitive somatic mutations, including *EGFR*-L858R and *EGFR*-19Del, are parallel to each other between CSF and matched PLA. In addition, the more frequent resistance gene *TP53* was found 4 (4/9, 44%) in CSF cfDNA, much higher than that in PLA (2/9, 22%). Moreover, *CDK4* (17%), *CDKN2A* (11%), *MET*, *SOX2*, *TERT*, *BRAF*, and *PIK3CG* (which have the same 6%) were detected exclusively in CSF (Fig. 4).

**Fig. 5:**
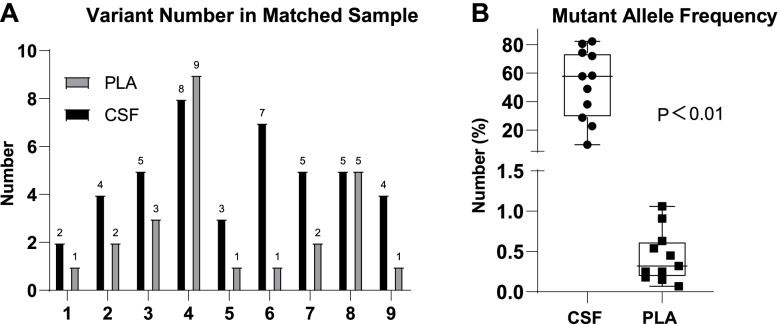
The amounts of variant genes and MAFs in matched CSF-PLA samples. **A** Difference in the amounts of variant genes detected in CSF and matched PLA. **B** MAF in CSF cfDNA and matched samples were analysed by Fisher’s exact test. *P* < 0.05 was deemed statistically significant. MAF: mutant allele frequency. CSF: cerebrospinal fluid. PLA plasma

The number of variant genes was more multiple in CSF than in matched PLA samples (Fig. 5A). Most importantly, all the MAFs in CSF cfDNA samples were significantly higher than those in plasma, the average MAFs of CSF cfDNA and plasma were 52.09 and 0.44%, respectively, and there was a statistically significant difference (*p* < 0.01) (Fig. 5B).

## Discussion

The expression of *EGFR* components is frequent in LUAD and makes *EGFR* an attractive anticancer target. Patients who share *EGFR* mutations could benefit from EGFR-TKIs and show a markedly better prognosis, but LM is still a devastating complication showing a dismal prognosis. Although significant projects are underway to create a comprehensive map of all the responsible genes involved in the evolution and dissemination of intracranial cancer, the driver genes in intracranial tumours remain unclear. NGS panels can detect variants in multiple relevant genes [6, 7], which provides a sufficient method to characterize resistance mechanisms. In our study, NGS was utilized in 45 CSF samples and revealed the genetic landscape of LM-LUAD. Primary sensitive mutations of *EGFR*-L858R or *EGFR*-Del19 were present in 31 (69%) patients, T790M was detected in 4 (9%) patients, *TP53* was present in 25 (56%) patients, and coexisting *TP53* and *EGFR* were found in 22 (49%) CSF specimens. *TP53* mutation is considered a poor prognostic factor, and cooccurring *TP53/EGFR* could affect the efficacy of EGFR-TKI in LUAD, leading to resistance to EGFR-TKI therapy and thus reducing OS [8, 9]. In our research, the subgroup with coexisting *TP53/EGFR* showed shorter OS than the opposing group (*p* = 0.04). This result was in agreement with the previous literature [8, 10], which reported that the concurrent is associated with poor outcomes. Molecular-level demonstrated that [11] *TP53* co-mutations are relatively frequent in these selected patients and exhibit this concurrent is associated with adverse outcome of crizotinib, showing significantly shorter progression-free survival (PFS) [8]. In our findings, the second most common coexisting gene mutation identified was *ALK* fusion together with *TP53*, which was discovered in 2/45 (4.4%) patients who had an OS and PFS of 2 and 7.5 months on crizotinib, respectively. Taking the limited number into consideration, there was no comparison of OS benefit results in our study.

The expression of *CDKN2A* correlated with poor prognosis and reduced patient survival [6, 12], and *CDKN2A* was identified in 9/45 (20%) patients in our research. However, this known genetic alteration is not correlated with a worse prognosis of patients with such an alternation (*p* = 0.2). Genetic heterogeneity among different anatomical regions and even in single cancer samples [11] may have led to the other results shown in our study. Extensive research is still needed to further elucidate this alternation. Gene signatures of *CDK4*, *PIK3CG*, or *PIK3CA* mutations were associated with poor survival [11, 13]; in our research, *CDK4* was identified in 8 (18%) patients, and *PIK3CG* was identified in 4 (9%) patients.

Further cohort analysis of *EGFR* mutation LM-LUAD patients identified the top 10 genes presented in each subgroup. Gene signatures consisting of *CDK4*, *CDKN2A*, *PIK3CG*, or *PIK3CA* mutations were more frequently identified in cohort 1, and all these genes are associated with poor survival [11, 13]. Unlike the genes discovered in cohort 1, *MET*, *YES1*, and *FGF3/FGF4* copy number amplifications were more likely present in cohort 2. In the context that the genomic features of tumours will guide therapeutic approaches, unique genomic alternation will be fundamental to the correct molecular diagnosis and treatment. Considering the findings in cohort one, it follows that targeting the *CDK4*, *PIK3CG*, or *PIK3CA* signalling pathways may help treat leptomeningeal metastases.

*EGFR* mutation (EGFRm) T790M was identified in 15% of patients in cohort one, which is much lower than the most common cause of acquired resistance of patients receiving EGFR-TKI treatment. An underlying reason might be that extracranial T790M status is not representative of intracranial conditions. The previous findings that T790M has a lower frequency intracranially further supported this assumption [5]. Shang et al. reported that in addition to the T790M mutation, *EGFR* amplification is another acquired drug resistance mechanism to icotinib/gefitinib [14]. *EGFR* amplification was detected at 23% (6/26) in cohort 1 and 50% (4/8) in cohort two, which partly accounted for the progressive disease of LM.

In this study, we characterized the genomic alteration of cfDNA in the CSF of LM-LUAD and compared it to matched PLA samples. We demonstrated that sensitive somatic mutations, including *EGFR*-L858R and *EGFR*-19Del, are parallel between CSF and matched PLA. Moreover, *CDK4*, *CDKN2A*, *MET*, *SOX2, TERT*, *BRAF*, and *PIK3CG* were detected exclusively in CSF, which will provide potentially clinically informative alterations for intracranial lesions. Importantly, we demonstrated that CSF cfDNA has a significantly higher MAF than plasma for CNS oncogenic alterations. This result is consistent with previous research showing that CSF demonstrates the relevant modifications of the gene in intracranial tumours and should be recommended as liquid biopsies to assess the evolution of CNS tumours [15]. However, due to the limited sample size in our study, research on larger sample sizes is still needed to distinguish our results from the background of random mutation.

It is known that [16, 17] brain metastasis samples share mutations that differ from the primary tumour, suggesting that intracranial metastasis lesions are more similar to one another than to the extracranial samples and suggesting that conferring CSF is a potential approach to detect lesions in the brain. The findings in our study suggest potential genomic alterations present in leptomeninges metastases. They may shed light on the divergence of therapeutic response in some cases, helping to select the optimal treatment dictated by the molecular characteristics of brain cancer.

Altogether, we identified gene alterations that may contribute to the metastatic process of leptomeningeal, and the analysis of CSF cfDNA should be another strategy to monitor intracranial tumour evolution when tissue is scant.

## Data Availability

The datasets used and/or analyzed during the current study are available from the corresponding author on reasonable request.
